# A mixed-methods quasi-experimental evaluation of a mobile health application and quality of care in the integrated community case management program in Malawi

**DOI:** 10.7189/jogh.09.010811

**Published:** 2019-06

**Authors:** Simone Peart Boyce, Florence Nyangara, Joy Kamunyori

**Affiliations:** 1 ICF, Atlanta, Georgia, USA; 2 ICF, Rockville, Maryland, USA; 3 US Pharmacopeia, Rockville, Maryland, USA; 4 ICF, Washington, DC, USA; 5 John Snow Inc., Pretoria, South Africa

## Abstract

**Background:**

The use of mobile health (mHealth) technology to improve quality of care (QoC) has increased over the last decade; limited evidence exists to espouse mHealth as a decision support tool, especially at the community level. This study presents evaluation findings of using a mobile application for integrated community case management (iCCM) by Malawi’s health surveillance assistants (HSAs) in four pilot districts to deliver lifesaving services for children.

**Methods:**

A quasi-experimental study design compared adherence to iCCM guidelines between HSAs using mobile application (n = 137) and paper-based tools (n = 113), supplemented with 47 key informant interviews on perceptions about QoC and sustainability of iCCM mobile application. The first four sick children presenting to each HSA for an initial consultation of an illness episode were observed by a Ministry of Health iCCM trainer for assessment, classification, and treatment. Results were compared using logistic regression, controlling for child-, HSA-, and district-level characteristics, with Holm-Bonferroni-adjusted significance levels for multiple comparison.

**Results:**

HSAs using the application tended to assess sick children according to iCCM guidelines more often than HSAs using paper-based tools for cough (adjusted proportion, 98% vs 91%; *P* < 0.01) and five physical danger signs – chest in-drawing; alertness; palmar pallor; malnourishment; oedema (80% vs 62%; *P* < 0.01), but not for fever (97% vs 93%; *P* = 0.06), diarrhoea (94% vs 87%; *P* = 0.03), and three danger signs – not able to eat or drink; vomits everything; has convulsions (88% vs 79%; *P* = 0.01). Across illnesses and danger signs, 81% of HSAs using the application correctly classified sick children, compared to 58% of HSAs using paper-based tools (*P* < 0.01). No differences existed for their treatment (*P* = 0.27). Interview respondents corroborated these findings that using iCCM mobile application ensures protocol adherence. Respondents noted barriers to its consistent and wide use including hardware problems and limited resources.

**Conclusion:**

Generally, the mobile application is a promising tool for improving adherence to the iCCM protocol for assessing sick children and classifying illness by HSAs. Limited effects on treatments and inconsistent use suggest the need for more studies on mHealth to improve QoC at community level.

Efforts to reduce mortality among children under five years of age have led to endeavors to improve community-level access to life-saving interventions to treat conditions responsible for majority of deaths in this age group [1]. Based on the World Health Organization (WHO) Integrated Management of Childhood Illness (IMCI) guidelines, integrated community case management (iCCM) is a proven, community-based strategy for managing, assessing, classifying, and treating common childhood illnesses (malaria, diarrhoea, and pneumonia) [2]. Under iCCM, community health workers (CHWs) are trained to identify illness and treat or refer sick children, thereby extending the reach of the public health system by leveraging these low-skilled front-line workers to populations with limited access to primary health care facilities [1]. Although iCCM can help save lives, reviews are mixed about the quality of care (QoC) provided by CHWs. Some argue that low educational levels and limited clinical background of CHWs decreases QoC, while others observe that better trained, supported, and supervised CHWs advance QoC since they offer timely services and more personalized care [3-7].

The rise in the availability of mobile phones in low- and middle-income countries in the last decade has increased the use of mobile technology to support health workers. Among other uses, mobile technology applications have been developed to provide assistance to health workers as decision support and adherence tools and to improve communication in the health care system—for referrals, medical supplies availability, and client outreach [8-12].

Although electronic health (eHealth) through the use of mobile phones – known as mHealth – is perceived to have great value in increasing efficiencies and reducing the burden of paper-based systems for health workers, not enough evidence from evaluations of eHealth implementations exists to properly guide and make an investment case for scale up; in fact, if improperly used, technology may have minimal impact on improving patient outcomes and only divert valuable resources [13]. The limited evidence available suggests that decision support tools can improve classification of illnesses, promote adherence to the IMCI protocol, and result in treatment with proper drug dosage [10,11]; however, these studies are often small and localized, and have tended to focus on mHealth for health workers in health facilities, with none explicitly addressing case management of childhood illnesses at the community level by CHWs [9,10]. As such, evidence of mHealth technologies improving QoC at the community level is needed in order to make the case for their use and scale up [13].

## The iCCM Program in Malawi

The under-five child mortality rate in Malawi has been decreasing steadily, from 234 deaths per 1000 live births in 1992 to 64 deaths per 1000 live births in 2015 [14]. Malaria, diarrhoea, and pneumonia account for approximately half of the under-five childhood deaths [15,16]. In 2008, Malawi introduced iCCM services, delivered by CHWs called health surveillance assistants (HSAs) through village clinics. HSAs are the peripheral cadre of health workers in the Ministry of Health (MOH), providing iCCM services in hard-to-reach areas (defined as more than five kilometers from a health facility or the presence of a physical barrier to a health facility) to children aged 2–59 months [17]. Their responsibilities include assessing, classifying, and treating children who present with common childhood illnesses—uncomplicated cases of fever, cough with fast breathing, diarrhoea, and eye infections. HSAs also identify signs of severe illnesses and refer these children, and children with illness that they cannot treat, to a nearby health facility.

From 2013 to 2017, Save the Children and its partners supported the MOH to implement iCCM in eight districts in Malawi with support from WHO through the Rapid Access Expansion (RAcE) program: Dedza, Likoma, Lilongwe, Mzimba North, Nkhata Bay, Ntcheu, Ntchisi, and Rumphi. In 2014, D-tree International developed a mobile application to help improve QoC provided by HSAs. The application incorporates the national iCCM protocol and guides HSAs through the sick child assessment and classification process and recommends a treatment plan. After registering the sick child in the application, the application guides the HSA through a series of questions for the caregiver; based on the caregiver’s responses, the application determines the illness and corresponding treatment and health education advice for the child. As part of the assessment, the application includes a stopwatch to assist the HSA in counting breaths per minute to determine respiratory rate. The iCCM application was piloted in four RAcE districts: Dedza, Mzimba North, Ntcheu, and Ntchisi.

HSAs were trained in iCCM according to national protocols by MOH trainers. HSAs in the iCCM mobile application pilot districts received an additional two-and-a-half days of training by D-tree on using the application as a decision-support tool. A select few HSAs in the intervention group received additional training on the application as “super users” to assist other HSAs with troubleshooting basic user issues.

We report findings from a mixed-methods evaluation of a decision-support mHealth application for HSAs to improve QoC compared to HSAs who use paper-based iCCM tools to manage childhood illnesses among children under five years of age.

## METHODS

This was a mixed-method quasi-experimental study with intervention and comparison groups and key informant interviews (KIIs) on stakeholders’ perceptions about QoC and the implementation and potential scale-up of the mobile application.

### Sample size and selection

The sample size, with an expected 1024 sick child observations, was calculated to detect an 11% point difference in HSA adherence for treatment to the iCCM protocol across the two groups, with 80% power and 5% significance. This difference was estimated from a recent study conducted in Malawi that determined protocol adherence of 73% and 62% among users and non-users of a mobile application, respectively [18]. Sample size was calculated using a design effect of 1.75—calculated from an interclass coefficient of 0.25 with a cluster size of four sick child visits per HSA—and then inflated by 3% for possible nonresponse or low-case load among some HSAs [19-21].

**Figure 1:**
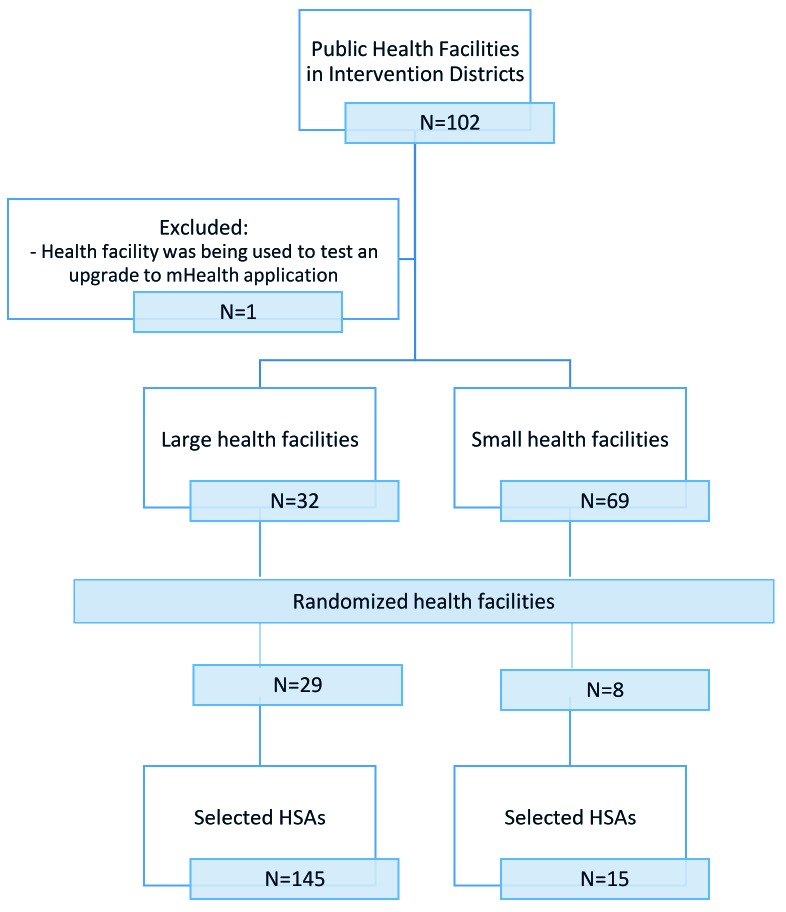
Sampling strategy for HSAs in intervention districts. mHealth – mobile health, HSA – health surveillance assistant.

**Figure 2:**
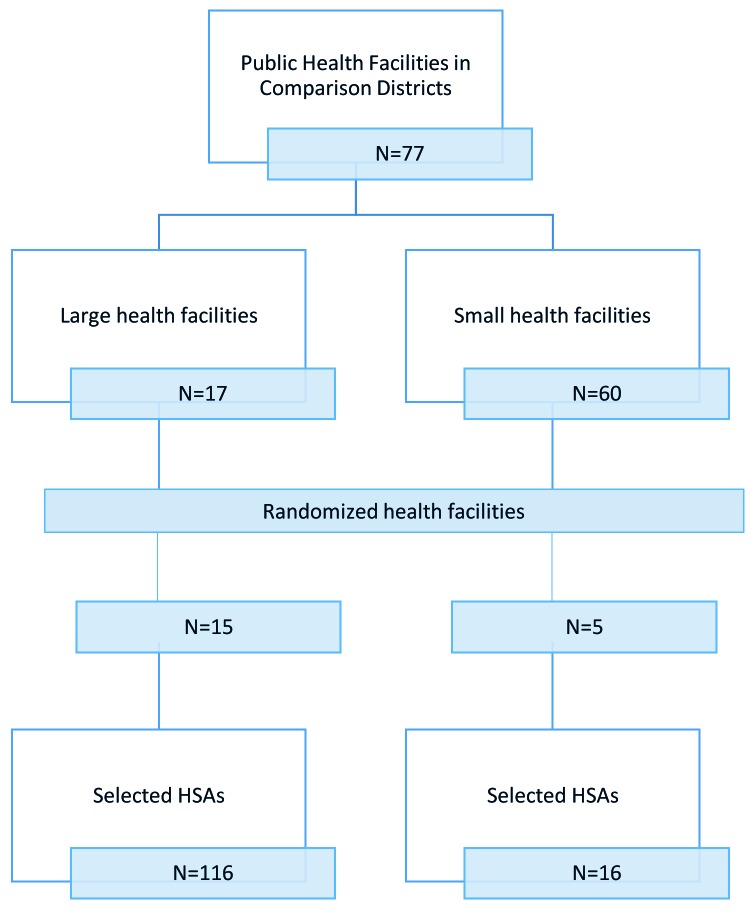
Sampling strategy for HSAs in the comparison districts. HSA – health surveillance assistant.

**Figure 3:**
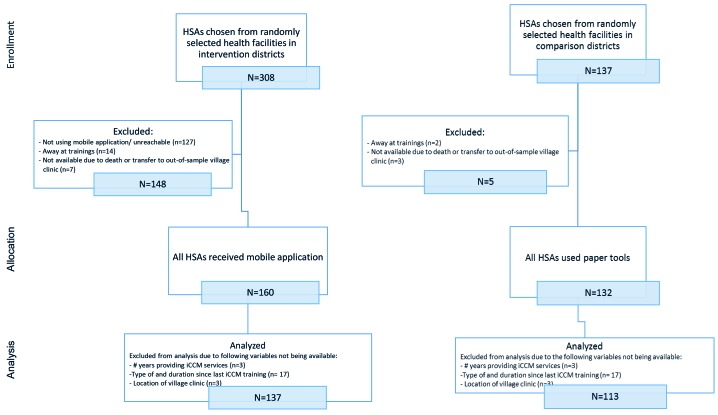
Participant flow of HSAs in intervention and comparison districts. iCCM – integrated community case management, HSA – health surveillance assistant.

The sampling frame included all trained and active HSAs (493 in the intervention group and 306 in the comparison group) working in the RAcE-supported districts. We anticipated selecting 256 HSAs reporting to randomly selected health facilities located in the RAcE-supported districts using a stratified random sample by size of facility. Large facilities – defined as facilities that supervise at least six HSAs – were sampled disproportionately, with approximately 75% of the selected facilities from this stratum and 25% of the selected facilities from the small stratum. The intervention group included 37 health facilities located in the four RAcE pilot districts (**Figure 1** and **Figure 2**). The comparison group included 20 health facilities in the three of the remaining RAcE districts – excluding Likoma. Only six HSAs were trained in Likoma, a small island in the middle of Lake Malawi. We excluded Likoma from the study due to the small number of trained HSAs and additional expense of travel. All HSAs from the sampled facilities were included in the comparison group, and only those using the mobile application from the intervention group. During fieldwork, some HSAs were found to be ineligible, away at trainings, or had died or transferred to an out-of-sample village clinic. The study sample included 160 intervention HSAs and 132 comparison HSAs (**Figure 3**).

### Data collection

During data collection, teams visited selected village clinics and assessed the first four children 2-59 months of age presenting to the HSA for an initial consultation on their current illness. Severely ill children who needed urgent referral to a health facility were excluded from the study.

Forty-seven KIIs were conducted with stakeholders representing all levels of the iCCM implementation system: the national IMCI coordinator; an IMCI monitoring and evaluation (M&E) officer; two D-tree staff members; a Save the Children M&E staff member; the district IMCI and RAcE program coordinators from all study districts; and two senior HSAs (SHSAs) and caregivers per district. SHAAs and caregivers were selected as follows: in each district, two facilities were randomly selected from which the SHSA reporting to each selected facility and one caregiver from each selected facility were interviewed. If more than one SHSA reported to a selected facility, one was selected at random to interview during the site visit.

All HSAs were interviewed to provide context for study findings. The interview questionnaire included questions regarding the HSA’s demographics, last initial or refresher iCCM training, typical data collection, case management and referral processes, as well as comfort with mobile technology and attitude toward the mobile scale-up (intervention group HSAs only). All interview guides were developed in English, with the caregiver guide translated into Chichewa (local language). Study authors conducted all national-level interviews, and the data collection teams conducted interviews with district- and facility-level staff, HSAs, and caregivers during the fieldwork visits.

Fifteen teams consisting of observers – nurses with clinical training in child health and IMCI – and evaluators – MOH iCCM trainers – collected the data, coordinated by the Centre for Agricultural Research and Design of the Lilongwe University of Agriculture and Natural Resources. To observe the assessment, classification, and treatment of children, observers used a case observation checklist based on tools from a previously conducted HSA QoC study in Malawi that used the WHO Heath Facility Survey checklist questionnaire [19]. The tool was updated to match the current iCCM protocol in Malawi and adapted for this study. HSAs were advised to indicate their treatment of the illnesses as if no stockouts of the necessary drugs existed. To ease the burden on sick children and caregivers, the evaluators simultaneously observed the sick child assessment by using an examination form based on caregiver responses to questions from the HSA and then used the assessment and any follow-up questions to independently classify and decide treatment for the child. The evaluator classifications and treatment decisions were used as the gold standard.

All enumerators participated in a five-day training and achieved 80% concordance with evaluators on two iCCM assessments using iCCM training videos that simulated assessment, classification, and treatment of a sick child. Data collection instruments and procedures were pretested and adapted to local conditions. The survey was piloted in village clinics external to the study to practice data collection under conditions that resembled those of the actual survey. Enumerators captured data in the field using tablet computers. All data were uploaded daily and checked for missing values and inconsistencies.

Enumerators did not collect data from village clinics where they normally worked to minimize bias, particularly from influencing HSA behavior and performance. Most HSAs do not open their village clinic every day, consequently they were informed of the site visit date to ensure their availability. In some instances, data collection teams returned to a village clinic if not enough sick children sought care from the HSA on the day of the previous visit. Written consent for participation in the study was obtained from HSAs and caregivers of the sick children.

### Data analysis

We conducted statistical analyses using weighted data to account for differential probabilities of selection and adjusted for clustering at the HSA level (Appendix S1 in **Online Supplementary Document**). Analysis was intention to treat. We used descriptive statistics to summarize sample characteristics and reported weighted percentages or means and 95% confidence intervals. We used logistic regression that included child-, HSA-, and district-level characteristics as covariates, and reported predicted probabilities for outcomes. Table S2 in **Online Supplementary Document** describes the outcome variables [19,22]. Covariates included child sex and age, HSA sex and educational level, tenure providing iCCM services as an HSA, type (initial/refresher) and duration since the last iCCM training, case load during the rainy season, village clinic location, access to an improved water source, and the median educational attainment of women. District-level data were derived from the Malawi Millennium Development Goals Endline Survey 2014 and the Malawi Demographic and Health Survey 2010 [23,24]. A Malawi Demographic and Health Survey had been completed in 2016, but district-level data had not yet been released by time of this study.

Access to improved water source was used to control for differences in district-level infrastructure. In retrospect, access to electricity may have been a better control because of the need to charge the mobile phones, but access to electricity varies widely between Lilongwe and the other districts. Our sample included HSAs from Lilongwe that were employed in rural Lilongwe only.

We restricted the overall sample to include only HSAs (n = 137 in the intervention group, n = 113 in the comparison group) and the corresponding sick children seen (n = 987) with non-missing data on the covariates. Characteristics of HSAs and sick children did not differ between the overall and restricted samples (data not shown). One exception was that the HSAs in the restricted sample tended to have completed more schooling (71% in the overall sample, compared to 75% in the restricted sample; *P* = 0.002).We used the Wald test to assess the comparability between the HSAs and children in the intervention and comparison groups. Two-tailed tests with Holm-Bonferroni adjusted significance levels for multiple comparisons indicated statistical significance. All data cleaning and analyses were conducted using Stata, version 14 (Stata Corp., College Station, TX, USA) for the quantitative data and ATLAS.ti (ATLAS.ti Scientific Software Development GmbH, Berlin, Germany) for the qualitative data—to identify themes on perspectives of stakeholders regarding mobile application in Malawi.

Ethical approval was obtained from the Institutional Review Boards of ICF and the Malawi MOH National Health Sciences Research Committee.

## RESULTS

HSAs in the intervention and comparison districts tended to be similar, however, more intervention HSAs (34%) had received an initial iCCM training more recently than those in the comparison group (11%) (*P* = 0.034) (**Table 1**). Almost 20% of HSAs in the intervention districts were not using the application on the day of the sick child visit. Reasons for not using the application included the mobile application being time-consuming or a non-functioning, lost, stolen, or out of power phone.

**Table 1:** Comparison of HSA characteristics in districts Using the iCCM mobile application and paper tools

Characteristics	iCCM application (N = 137)			Paper tools (N = 113)			*P*-value
**N**	**Weighted %**	**95% CI**	**N**	**Weighted %**	**95% CI**
Age (years, mean)	137	36.0	33.8, 38.2	110	38.0	37.0, 39.0	0.113
Gender:							
-Female	39	35.1	19.0, 55.5	25	14.7	7.6, 26.6	**0.042**
-Male	98	64.9	44.5, 81.0	88	85.3	73.4, 92.4	
Highest level of education completed:							
-At most two years of secondary school	37	24.4	13.4, 40.4	34	26.5	15.7, 41.1	0.828
-Secondary school or higher	100	75.6	59.6, 86.6	79	73.5	58.9, 84.3	
Years providing iCCM services as HSA (mean)	137	5.4	4.3, 6.6	113	4.7	4.2, 5.2	0.273
Lives in village clinic catchment area	118	76.4	59.3, 87.8	68	77.3	57.2, 89.7	0.930
Sick children seen per day (mean):							
-Rainy season	137	16.3	12.1, 20.6	113	19.1	14.9, 23.2	0.361
-Dry season	137	9.1	6.9, 11.3	113	10.0	7.6, 12.4	0.592
Village clinic located in room not attached or not next to home	55	54.6	38.8, 69.4	85	80.0	70.3, 87.1	**0.007**
Days in past 7 days operate village clinic (mean)	134	3.5	2.7, 4.4	113	3.5	2.5, 4.5	0.930
Hours in past 7 days operate village clinic (mean)	134	30.6	21.6, 39.6	113	32.3	8.2, 56.3	0.896
Primary iCCM protocol used*							
-Sick child recording form	26	16.8	9.6, 27.8	72	46.9	20.6, 75.0	**0.039**
-Village clinic register	69	60.1	39.8, 77.4	113	100.0	100.0, 100.0	**<0.001**
-iCCM mobile application	109	79.1	63.4, 89.2	0	0.0	0.0, 0.0	**<0.001**
Items currently included in drug box†	137	9.2	8.7, 9.6	113	8.9	8.5, 9.3	0.349
Items stockout that lasted 7 days or moreǂ	137	1.7	1.1, 2.2	113	2.0	1.4, 2.7	0.358
**Training, supervision, and mentoring**							
Most recent iCCM training:							
-Initial	53	34.0	16.8, 56.9	18	11.7	5.5, 23.1	**0.034**
-Refresher	84	66.0	43.1, 83.2	95	88.3	76.9, 94.5	
Months since most recent iCCM training (median, interquartile range)	137	4.4	2.4, 18.4	113	2.4	2.4, 15.4	0.508
Tools trained on in most recent iCCM training (mean)§	137	2.9	2.9, 3.0	113	2.9	2.9, 3.0	0.981
Days report to health facility in past month (mean)	137	5.8	4.8, 6.8	113	7.1	5.5, 8.6	0.170
Supervisory visits in past 3 months (mean)	137	1.2	1.0, 1.3	113	1.0	0.8, 1.3	0.330
Most recent supervisory visit by senior HSA (%)	93	69.5	47.4, 85.2	57	60.1	41.0, 76.5	0.494
Tasks conducted during most recent supervisory visit (mean)	137	5.6	5.2, 6.1	113	4.7	3.6, 5.8	0.138
Mentor visits in past 3 months (mean)	136	0.8	0.6, 1.1	113	0.7	0.3, 1.0	0.433
Tasks conducted during most recent mentor visit (mean)¶	137	3.0	2.2, 3.8	113	2.0	1.0, 3.1	0.160

iCCM – integrated community case management, HSA – health surveillance assistant

*Categories are not mutually exclusive because HSA may use multiple guides.

†Drug box should include 11 items: LA (1 × 6 and 2 × 6 blister packets), rapid diagnostic test, rectal artesunate, amoxicillin/cotrimoxazole, oral rehydration solution, zinc, paracetamol, eye antibiotic, timer, and gloves.  ‡Nine items included for stockout: LA (1 × 6 and 2 × 6 blister packets), rapid diagnostic test, rectal artesunate, amoxicillin, oral rehydration solution, zinc, paracetamol, and eye antibiotic.

§The tools include sick child recording form, village clinic register, and referral slip.

Seven possible tasks are included: reviewing village clinic register, checking supplies and equipment levels, using a supervision checklist, administering a case scenario, observing management of a sick child, meeting with village committee members, and giving feedback on iCCM activities.

¶Four possible tasks are included: using a mentoring checklist, observing management of a sick child, demonstrating how to care for a sick child or identify danger signs, and giving feedback on case management skills.

During the sick child visit, HSAs initially asked caregivers for the primary reasons for the visit. Differences existed in care-seeking behavior of caregivers (Table 2). Despite these differences, however, no statistically significant differences existed between sick children in the intervention and comparison districts for the majority of illness classifications, based on the gold-standard of the evaluator (**Table 3**).

**Table 2:** Characteristics and presenting complaints of observed sick children seen by HSAs in districts using the iCCM mobile application and paper tools

Characteristics	iCCM mobile application (N = 535)			Paper tools (N = 452)			*P*-value
**N**	**Weighted %**	**(95% CI)**	**N**	**Weighted %**	**(95% CI)**
Age (months; mean)	535	23.5	21.1, 25.8	452	23.3	21.5, 25.2	0.943
Gender:							
-Female	275	53.2	46.0, 60.4	237	58.1	51.0, 64.9	0.347
-Male	260	46.8	39.6, 54.0	215	41.9	35.1, 49.0	
Presenting complaint of observed sick children as reported by caregiver*							
Fast or difficult breathing	7	0.7	0.3, 1.5	23	6.4	3.4, 11.8	**<0.001**
Cough	367	69.1	62.4, 75.1	309	62.9	55.7, 69.6	0.200
Pneumonia	1	0.1	0.0, 0.7	21	5.2	2.7, 9.9	**<0.001**
Diarrhoea (loose stools)	126	20.9	15.9, 26.9	107	30.3	23.5, 38.0	0.043
Fever	308	61.5	56.0, 66.8	294	65.2	57.9, 71.9	0.421
Malaria	2	1.0	0.2, 6.0	52	7.6	4.9, 11.5	**0.030**
Convulsions	1	0.1	0.0, 0.7	3	0.3	0.1, 1.0	0.307
Sleepy or unconscious	0	0.0	0.0, 0.0	8	0.9	0.5, 1.6	**0.002**
Difficulty drinking or feeding	11	3.6	1.5, 8.6	14	2.5	1.0, 6.1	0.575
Vomiting	58	12.4	8.2, 18.5	59	13.3	8.4, 20.3	0.837
Red eyes	37	6.2	3.6, 10.5	18	3.9	1.7, 8.6	0.350
Other problem mentioned	46	12.1	7.6, 18.6	50	9.3	5.7, 14.9	0.441

iCCM – integrated community case management, HSA – health surveillance assistant

*Categories are not mutually exclusive as caregivers may report multiple complaints.

**Table 3:** Classification of observed sick children seen by HSAs in districts using the iCCM Mobile Application and Paper Tools, Based on Gold Standard Re-examination

Characteristics	iCCM mobile application (N = 535)			Paper tools (N = 452)			*P*-value
**N**	**Weighted %**	**95% CI**	**N**	**Weighted %**	**95% CI**
Cough with fast breathing	99	21.5	15.7, 28.8	73	21.7	15.3, 29.7	0.980
Fever:							
-Less than 7 days	330	67.1	61.6, 72.1	309	70.8	64.6, 76.3	0.356
-7 days or more	5	1.3	0.3, 5.3	10	3.0	1.3, 6.9	0.319
Diarrhoea:							
-Less than 14 days and no blood in stool	127	21.0	16.2, 26.7	96	28.1	21.7, 35.5	0.108
-14 days or more	7	0.7	0.3, 1.5	1	0.1	0.0, 0.8	0.081
-Blood in stool	13	2.1	0.8, 5.3	8	2.8	0.9, 8.2	0.694
Red eyes:							
-Less than 4 days	26	5.1	2.6, 9.6	18	5.9	2.9, 11.4	0.764
-4 days or more	3	2.3	1.0, 5.3	5	0.5	0.2, 1.2	**0.015**
-Visual problem	0	0.0	0.0, 0.0	1	0.1	0.0, 0.8	0.318
Chest indrawing	11	1.9	0.7, 5.2	7	1.7	0.5, 6.0	0.900
Vomits everything	3	1.1	0.2, 5.7	3	0.3	0.1, 1.0	0.219
Palmar pallor	3	0.3	0.1, 0.9	2	0.2	0.1, 0.9	0.722
MUAC tape:							
-Red	0	0.3	0.1, 0.9	3	0.3	0.1, 1.0	0.921
-Yellow	3	0.3	0.1, 0.9	10	3.0	1.1, 8.2	**0.003**
Convulsions	1	0.1	0.0, 0.7	4	0.4	0.2, 1.2	0.190
Not able to drink or feed anything	1	0.1	0.0, 0.7	3	0.3	0.1, 1.0	0.307
Very sleepy or unconscious	3	0.0	0.0, 0.0	1	0.1	0.0, 0.8	0.318
Swelling of both feet	0	0.0	0.0, 0.0	2	1.2	0.2, 7.0	0.275
Other problems, refer	57	12.3	7.9, 18.7	60	11.4	7.3, 17.4	0.800

CI – confidence interval iCCM – integrated community case management, HSA – health surveillance assistant, MUAC – mid-upper arm circumference

### Assessment, classification, treatment, and counseling

HSAs using the mobile application tended to assess sick children according to the iCCM protocol more often than HSAs using paper-based tools for certain conditions (**Table 4**). In particular, a higher percentage of children seen by intervention HSAs were assessed for cough (*P* < 0.001) and the five danger signs (*P* < 0.001). There were no statistically significant differences between the groups for children assessed for diarrhoea (*P* = 0.026), malaria with rapid diagnostic tests (*P* = 0.507), fever (*P* = 0.056), fast breathing through counting of respiratory rates (*P* = 0.462), and the three general danger signs (*P* = 0.009).

**Table 4:** Predicted probabilities of the correct assessment for illnesses of observed sick children seen by HSAs in districts using the iCCM mobile application and paper tools*

Symptoms		iCCM mobile application		Paper tools		*P*-value†
**N**	**Weighted %**	**95% CI**	**Weighted %**	**95% CI**
Children checked for presence of cough	987	97.9	96.6, 99.2	90.7	85.5, 95.9	**0.001**
Children checked for presence of diarrhoea	987	93.9	90.8, 96.9	87.4	82.1, 92.6	0.026
Children checked for presence of fever	987	96.7	94.4, 99.0	92.6	87.6, 97.6	0.056
Children with cough assessed for presence of fast breathing through counting of respiratory rates	716	97.1	94.3, 99.8	95.7	92.6, 98.9	0.463
Children with cough assessed for the presence of fast breathing in which HSA counted respiratory rate within ± 3 breaths of gold standard (N = 699)	699	84.8	81.3, 88.3	86.6	82.2, 91.0	0.488
Children with fever assessed for malaria with rapid diagnostic test	652	83.8	73.3, 94.2	88.6	81.9, 95.3	0.507
Children assessed for three general danger signs	987	87.6	83.6, 91.6	78.6	73.3, 84.0	0.009
Children checked if able to drink or eat anything	987	94.9	92.9, 97.0	89.4	86.0, 92.9	**<0.001**
Children checked if vomit everything	987	94.1	90.7, 97.6	91.1	86.9, 95.4	0.270
Children checked if have convulsions	987	92.8	90.7, 94.8	84.0	80.1, 87.9	**<0.001**
Children assessed for five physical danger signs	987	79.9	75.9, 84.0	61.7	55.0, 68.4	**<0.001**
Children checked for chest indrawing	987	94.6	92.8, 96.3	78.2	73.5, 82.9	**<0.001**
Children checked if sleepy or unconscious	987	98.6	97.0, 100.1	96.5	93.6, 99.5	**<0.001**
Children checked for palmar pallor	987	99.1	98.5, 99.8	89.6	84.6, 94.6	**<0.001**
Children checked for malnutrition with MUAC tape	987	86.3	82.9, 89.7	82.6	77.6, 87.6	0.182
Children checked if swelling of both feet	987	96.6	95.0, 98.2	85.9	80.9, 91.0	**<0.001**

iCCM – integrated community case management, HSA – health surveillance assistant, MUAC – mid-upper arm circumference

*Probabilities adjusted for child characteristics (age and gender), HSA characteristics (gender, highest education level, tenure as an HSA, type and duration since most recent iCCM training, patient case load, and village clinic location), and district characteristics (access to improved water source and median number of years of women’s education) using logistic regression with standard errors clustered at the HSA level.

†Compared against Holm-Bonferroni adjusted significance levels.

More than 80% of HSAs using the mobile application classified sick children across the common illnesses and danger signs similarly to the evaluator, compared to 58% of the comparison group (*P* < 0.001) (**Table 5**). HSAs, especially those using paper-based tools, tended to misclassify non-febrile children as febrile and failed to classify nourished children. No statistically significant differences between the two groups of HSAs for the illnesses were found (*P* = 0.025).

**Table 5:** Predicted probabilities of the correct classification of illnesses of observed sick children seen by HSAs in districts using the iCCM mobile application and paper tools*

Classification			iCCM Mobile application			Paper tools					
**N**	**Weighted %**	**95% CI**		**Weighted %**		**95% CI**			***P*-value†**	
Children whose classifications given by HSA match all classifications given by evaluator‡		987	80.7	76.4, 84.9		57.6		49.6, 65.6		**<0.001**	
Children classified by HSA in the three common illnesses (malaria [positive mRDT], diarrhoea, and cough with fast breathing) that match the evaluator classifications		987	91.3	87.6, 95.0		82.5		75.3, 89.8		0.025	
Malaria (positive mRDT)		987	99.7	99.1, 100.0		99.9		99.8, 100.0		0.392	
Diarrhoea	987		95.7		93.2, 98.2	91.4			86.3, 96.5		0.095	
Cough with fast breathing		987	95.6	92.9, 98.4		89.2	82.1, 96.2			0.055	

iCCM – integrated community case management, HSA – health surveillance assistant, MUAC – mid-upper arm circumference, mRDT – malaria rapid diagnostic test, CI – confidence interval

*Probabilities are adjusted for child characteristics (age and gender), HSA characteristics (gender, highest education level, tenure as an HSA, type and duration since most recent iCCM training, patient case load, and village clinic location), and district characteristics (access to improved water source and median number of years of women’s education) using logistic regression with standard errors clustered at the HSA level.

†Compared against Holm-Bonferroni adjusted significance levels.

‡ Classifications include diarrhoea, cough, fever, fast breathing, blood in stool, chest indrawing, convulsions, not eating or drinking, vomiting everything, red eye, red eye with visual problems, sleepy or unconscious, palmar pallor, foot swelling, and color on the MUAC tape.

Overall, children with the common illnesses received the correct treatment for their illnesses, regardless of the tool used to guide treatment (**Table 6**). Investigation by each illness showed that intervention HSAs tended to prescribe an antimalarial drug correctly to children with fever and positive malaria rapid diagnostic test (mRDT) more often (80%) than comparison HSAs (52%) (*P* < 0.001). Further investigation indicated no differences in treatment due to age band mixing. Instead differences were found in whether any treatment was offered for malaria. Of those HSAs incorrectly treating malaria, 67% (62% in comparison and 4% in intervention) failed to offer any treatment despite positive mRDT. Almost 90% of HSAs in the intervention districts correctly referred children with danger signs in need of a referral as compared to 71% of HSAs in comparison districts (*P* = 0.010). The majority of interview respondents corroborated these findings that use of the mobile application improves adherence to the protocol. One SHSA said; *“There are no short cuts…not allow HSAs to skip as the phone guides you step by step.*” However, one national stakeholder cautioned that adherence varies and the practical effect of the mobile application depended on the HSA's characteristics.

**Table 6:** Predicted probabilities of the correct treatment, referral, and counseling of children seen by HSAs in districts using the iCCM mobile application and paper tools

Treatment/ Referral/Counseling		iCCM mobile application							Paper tools					*P*-value†	
**N**		**Weighted %**		**95% CI**	**N**		**Weighted %**			**95% CI**	
**Treatment:**															
Children with cough and fast breathing, positive mRDT, or diarrhoea who are correctly prescribed all medications (antibiotic, antimalarial drug, or ORS and zinc) for their illnesses		223		69.9		62.5, 77.4	186		64.7			58.8, 70.6		0.267	
Children with cough and fast breathing who are prescribed an antibiotic correctly		73		70.8		67.9, 73.8	53		74.6			71.1, 78.2		0.147	
Children with fever and positive mRDT who are prescribed an antimalarial drug correctly		80		80.0		75.6, 84.5	89		51.8			47.0, 56.7		**<0.001**	
Children with diarrhoea who are prescribed ORS and zinc correctly		106		66.8		56.2, 77.5	78		68.7			60.1, 77.3		0.760	
Children without cough and fast breathing who would have left the HSA without having received an antibiotic		349		97.3		94.3,100.0	297		98.2			96.2, 100.0		0.561	
**Referral:**															
Children with danger signs needing referral who are referred		96		87.0		83.9, 90.1	88		70.8			57.0, 84.6		**0.010**	
**Counseling:**															
Children who need an antibiotic, ORS and zinc, or antimalarial drug who receive the correct first dose in presence of HSA		223		28.5		18.2, 38.9	186		35.5			25.6, 45.3		0.373	
Caregivers of children with cough and fast breathing, positive mRDT, or diarrhoea who are counseled on their illnesses		233		29.0		19.6, 38.4	204		46.4			33.3, 59.6		0.058	
Cough and fast breathing	79		58.7		53.1, 64.2			61		65.0	60.2, 69.8		0.155	
Diarrhoea	114		5.4		2.4, 8.3			89		23.0	8.0, 38.0		0.014	
Fever and positive mRDT	83		57.2		47.2, 67.2			93		64.7	51.3, 78.2		0.374	

iCCM – integrated community case management, HSA – health surveillance assistant, MUAC – mid-upper arm circumference, mRDT – malaria rapid diagnostic test, ORS – oral rehydration salts

*HSAs prescribed antimalarial drugs for less than 0.5 percent of children with fever and negative mRDT. Probabilities adjusted for child characteristics (age and gender), HSA characteristics (gender, highest education level, tenure as an HSA, type and duration since most recent iCCM training, patient case load, and village clinic location), and district characteristics (access to improved water source and median number of years of women’s education) using logistic regression with standard errors clustered at the HSA level.

†Compared against Holm-Bonferroni adjusted significance levels.

To assess whether caregivers received counseling on the correct administration of drugs, we focused on whether the HSA encouraged the caregiver to give the first dose of treatment to the child in his or her presence. Few children treated by HSAs in either group received their first dose of treatment at the village clinic. This low rate prompted us to explore whether this was remedied by HSAs counseling the caregiver on dosage, frequency, and duration of administering treatments; demonstrating treatment; and requiring caregivers to repeat the instructions for the treatment procedure. Fewer than half of all HSAs in either group, however, provided this support.

### Stakeholder perception of mobile application

Interviews with stakeholders in intervention districts and facilities indicated that HSAs liked the mobile application and generally found it easy to use. They described the mobile application as *“user friendly”* “*very logical”, and “requir(ing) HSAs to complete all steps in proper order.”* When asked to identify factors that have facilitated the adoption of the mobile application, respondents described a collaborative process to develop and roll-out the mobile application: D-tree led the development, with input from MOH and Save the Children, and supported all trainings. Other factors noted were recruitment of young HSAs who have higher uptake of technology; simplicity of assessment, classification, and treatment protocols; offering training, supervision, and opportunities to practice using the application; and the availability of “super users” to assist with troubleshooting basic issues.

When asked about problems HSAs encountered when using the mobile application, informants reported that some HSAs were not using the phones consistently because the application was time consuming; hardware and software problems related to malfunctioning phones, inadequate battery power for phones, or problems charging the phone; inadequate airtime and network coverage to sync data from the application; and inability of SHSAs to assist HSAs under their supervision with phone problems. While “super users” were available to assist HSAs, they were not always able to resolve issues, which then required requesting help from D-tree. This was corroborated by approximately 35% of contacted HSAs in intervention districts not using their mobile application or unreachable, resulting in their exclusion from the sample.

## DISCUSSION

HSAs who used the iCCM mobile application had higher rates of assessing and classifying sick children correctly, compared to HSAs using the paper-based tool, however, even the differences seen were marginally significant. No statistically significant differences were found between the two groups in classification and treatment of children with the three main iCCM illnesses and in counseling caregivers. Predicted probabilities of correct treatment and counseling were largely similar, which corroborates a previous study finding that there were no differences in treatment rates between CHWs using electronic and paper-based tools [25]. Overall, the results show that the mobile application can help HSAs to adhere to the protocol in terms of conducting assessments of sick children and classifying diseases. A study by Mitchell et al. found similar results in Tanzania, where adherence to the IMCI protocol used in health facilities was greater for health workers using an electronic IMCI tool than for those using a paper-based tool [10]. Indeed, there is a growing body of evidence that mHealth is a promising tool for shifting some health services tasks that require compliance with guidelines and protocols from clinicians to frontline health workers through the use of algorithm-based decision-support tools[26,27]. However, use of the mobile application was found to be insufficient to improve health outcomes through correct treatment and counseling caregivers.

The majority of stakeholder interviewees liked the mobile application and cited its user-friendliness, despite some challenges using the phones and syncing data. However, some HSAs indicated lack of consistent use of the mobile application because it was time-consuming or hardware or software problems with the phone.

DeRenzi et al., in a study assessing the feasibility of electronic IMCI (eIMCI) tools for improving pediatric care in Tanzania, found that a key factor for acceptability of the eIMCI application by clinicians was speed – a tool seen as taking too long to step through the protocol would be discarded [28]. This corroborates the finding that some HSAs stopped using the application because it was too time-consuming. In designing their application, DeRenzi et al. developed a tool that combined clinicians’ use of their experience and prescriptive elements of the protocol, resulting in the application being almost as fast as existing practice – where clinicians rarely consulted the paper tool and instead relied on their memory and training. It is not clear whether such a solution would be suitable in a community setting, where HSAs are generally poorly educated and trained.

Much of the literature refers to “technological” challenges when deploying mHealth interventions in low-resource settings, some of which align with the experiences cited by HSAs: poor mobile network connectivity; lack of or limited electricity to charge phones; lost or damaged phones; costs of handsets; and poor mobile phone maintenance [29]. The literature, however, tends not to expound on how these have been addressed; more attention is generally given to “big picture” issues, such as usability, training, national policies, and technical standards. As mHealth is increasingly considered as an effective tool for improving community health services in resource-constrained settings, additional discussion is warranted on these more mundane challenges of community-level interventions, as they remain important considerations for implementation and scale up [30].

A limitation of this study was the eligibility criteria in the intervention districts that included only HSAs using the mobile application. This provided incidental findings around HSAs’ acceptance and use of the mobile application but also potentially biases the results: HSAs who chose not to use the application may be ones who are weaker in the application of iCCM. However, the actual direction of the bias cannot be measured without understanding the characteristics of the HSAs not using the phone. Because this was not the focus of our study, we did not probe this; more qualitative studies should be conducted to determine why HSAs are not using the mobile application, and if there are correlations to any other factors.

The findings of this study should be viewed in light of certain limitations. First, the sick child assessments were conducted simultaneously by the HSA and the evaluator. This approach was strongly recommended by the MOH iCCM trainers during the study training due to concerns about burdening the sick children and caregivers, however it does not follow usual practice (observation followed by separate re-examination) for QoC evaluations. Additionally, the study was not part of the initial program design, so we were not able to randomize the intervention nor have a baseline assessment. Due to the lack of baseline assessment, we were unable to determine if any differences existed between the groups prior to the implementation of the mobile application such as district capacity. We used multivariate regression analysis to control for possible confounding factors related to the district, facility, and HSA observable characteristics that may bias our findings. Also, relying on observation of HSAs as they assess sick children has the potential to introduce bias if HSAs change their assessment habits to satisfy the observer, also referred to as the Hawthorne effect [31]. Since this approach was used for both intervention and comparison HSAs, this should affect both groups and minimize any bias in performance between the groups.

## CONCLUSION

Results of the study lend some support to the mobile application as a tool to improve adherence to the iCCM protocol for assessing sick children and classifying illness, especially for less-trained HSAs in hard-to-reach areas with severe shortages of trained health personnel. However, the lack of effect on treatment points to additional support required regarding adherence to the treatment protocol. Additionally, even the significant differences seen in assessment and classification were marginal. It is difficult, therefore, to conclusively say that mHealth for community-level decision support improves QoC; more studies of these technologies at this level are required to develop a solid evidence base.

## Additional material

Online Supplementary Document
