# Supplementary material for: A mixed-methods quasi-experimental evaluation of a mobile health application and quality of care in the integrated community case management program in Malawi
Source: J Glob Health. 2019 Jun 25;9(1):010811. doi: 10.7189/jogh.09.010811 (PMC6594718; doi:10.7189/jogh.09.010811)
Supplement: Online Supplementary Document [file jogh-09-010811-s001.pdf]

J Glob Health 2019; 9: 010811

Appendix S1: Calculating Sample Weights

Sample weights were calculated to guarantee the representativeness of the data given the cluster random design. Weights were calculated from the selection probabilities, equal to the reciprocal of the probability of selection into the sample by strata, or the sum of the number of HSAs supporting the facilities divided by the number of HSAs selected for inclusion in the evaluation by strata and study group (see Table S1). Sampling weights were adjusted for those that were not eligible—HSAs were deleted from the denominator of the sampling probabilities.

Table S1: Sample Weights, by Strata

	Facility Size	
Study Group	Large	Small
Intervention	1.16	10.85
Comparison	1.08	10.94

Table S2. List and Definition of Key Quality of Care Performance Indicators

Key Indicator	Definition
**Assessment**	
Children checked for presence of cough	Proportion of sick children observed whose caretakers were asked for presence of cough
Children checked for presence of diarrhoea	Proportion of sick children observed whose caretakers were asked for presence of diarrhoea
Children checked for presence of fever	Proportion of sick children observed whose caretakers were asked for presence of fever
Children with cough assessed for presence of fast breathing through counting of respiratory rates	Proportion of sick children observed with cough who had respiratory rate counted by HSA
Children with fever assessed for malaria with a rapid diagnostic test (mRDT)	Proportion of sick children observed with fever/history of fever assessed for malaria with a mRDT
Children checked for three general danger signs	Proportion of sick children observed who are checked for three general danger signs: not able to drink/BF/eat, vomits everything, has convulsions
Sick children assessed for five physical danger signs, overall and disaggregated by danger sign*	Proportion of sick children observed who are assessed for five physical danger signs: chest indrawing; sleepy or unconscious, palmar pallor; red on MUAC tape; swelling of both feet
Cases of children with cough assessed for the presence of fast breathing in which HSA counted respiratory rate within +/- 3 breaths of gold standard (iCCM trainer)	Proportion of cases of children observed with cough assessed for the presence of fast breathing in which HSA counted respiratory rate within +/- 3 breaths of gold standard (iCCM trainer)
**Classification**	
Children whose classifications given by HSA match all the classifications given by IMCI-trained clinician/evaluator	Proportion of sick children observed with validated classifications for whom classifications for the main symptoms given by HSA match all the validated classifications
Children whose classifications for the three common illnesses given by HSA match the classifications given by IMCI- trained clinician/evaluator	Proportion of sick children observed with validated classifications for whom classifications for three common illnesses (malaria [positive mRDT], diarrhoea and cough with fast breathing) given by HSA match the validated classifications
**Treatment**	

Key Indicator	Definition
Children with cough and fast breathing and/or positive mRDT and/or diarrhoea who are correctly prescribed all medications (antibiotic and/or antimalarial and/or ORS and zinc) for their illness(es) †	Proportion of sick children observed with validated classifications, not needing referral, with cough and fast breathing, positive mRDT and/or diarrhoea who are correctly prescribed an oral antibiotic, antimalarial and/or ORS and zinc, including correct dose, frequency and duration
Children with cough and fast breathing who are prescribed an antibiotic correctly†	Proportion of sick children observed with validated classifications, not needing referral, with cough and fast breathing who are correctly prescribed an oral antibiotic, including correct dose, frequency and duration
Children with fever and positive mRDT who are prescribed an antimalarial (ACT) correctly†	Proportion of sick children observed with validated classifications not needing referral who have positive mRDT who are correctly prescribed an antimalarial, including correct dose, frequency and duration
Children with fever and negative mRDT who are prescribed an antimalarial (ACT) †	Proportion of sick children observed with validated classifications and a negative mRDT result not needing referral or an antimalarial who have fever/history of fever but negative mRDT who are incorrectly prescribed an antimalarial
Children with diarrhoea who are prescribed ORS and zinc correctly†	Proportion of sick children observed, with validated classifications, not needing referral, with diarrhoea who are correctly prescribed ORS and zinc, including correct dose, frequency and duration
Children without cough and fast breathing who would have left the HSA without having received an antibiotic†	Proportion of sick children observed, with validated classifications, not needing referral, who do not need an oral antibiotic for cough and fast breathing who would have left HSA without antibiotic
Children who need an antibiotic, ORS and zinc, and/or antimalarial who receive the correct first dose in presence of HSA†	Proportion of sick children observed, with validated classifications, not needing referral, who need oral antibiotic, antimalarial and/or ORS and zinc and received the first dose of all needed drugs in presence of HSA
**Referral**	
Children with danger signs needing referral who are referred‡	Proportion of sick children observed with validated classifications needing referral due to the presence of one or more danger signs who were referred

Notes: iCCM: Integrated Community Case Management; HSA: Health Surveillance Assistant; MUAC: Mid-Upper Arm Circumference; mRDT: malaria Rapid Diagnostic Test; ORS: Oral Rehydration Salts; IMCI: Integrated Management of Childhood Illness; ACT: Artemisinin-based combination therapy

* Children 2-5 months only assessed for 3 physical danger signs (MUAC only assessed for children 6 months and older).

† Among children not presenting with danger signs and requiring referral

‡ Includes: 1) cough for 21 days or more; 2) diarrhoea for 14 days or more; 3) Blood in stool; 4) Fever for last 7 days; 5) Convulsions; 6) Child not able to drink or feed anything; 7) Red eye for 4 days or more; 8) Red eye with visual problems; 9) Chest in-drawing; 10)

Very sleepy or unconscious; 11) Palmar pallor; 12) Red on MUAC tape; 13) Swelling of both feet; 14) Other problem HSAs cannot treat

Adapted from:

Johns Hopkins University. Quality of Care Provided to Sick Children by Health Surveillance Assistants in Malawi: Final Report. 2009.

World Health Organization. Health Facility Survey Tool to Evaluate the Quality of Care Delivered to Sick Children Attending Outpatient Facilities: Using the Integrated Management of Childhood Illness Clinical Guidelines as Best Practices. Geneva, Switzerland: World Health Organization, 2001.
